# Indole-3-Acetic Acid Alters Intestinal Microbiota and Alleviates Ankylosing Spondylitis in Mice

**DOI:** 10.3389/fimmu.2022.762580

**Published:** 2022-02-04

**Authors:** Jun Shen, Lianjun Yang, Ke You, Tao Chen, Zhihai Su, Zhifei Cui, Min Wang, Weicong Zhang, Bin Liu, Kai Zhou, Hai Lu

**Affiliations:** ^1^ Department of Spine Surgery, The Fifth Affiliated Hospital, Sun Yat-sen University, Zhuhai, China; ^2^ Shenzhen Institute of Respiratory Diseases, Second Clinical Medical College (Shenzhen People’s Hospital), Jinan University, First Affiliated Hospital (Shenzhen People’s Hospital), Southern University of Science and Technology, Shenzhen, China

**Keywords:** ankylosing spondylitis, indole-3-acetic acid, inflammatory response, aryl hydrocarbon receptor, intestinal microbiota

## Abstract

Ankylosing spondylitis (AS) is a systemic, chronic, and inflammatory autoimmune disease associated with the disorder of intestinal microbiota. Unfortunately, effective therapies for AS are lacking. Recent evidence has indicated that indole-3-acetic acid (IAA), an important microbial tryptophan metabolite, can modulate intestinal homeostasis and suppress inflammatory responses. However, reports have not examined the *in vivo* protective effects of IAA against AS. In this study, we investigated the protective effects and underlying mechanisms through which IAA acts against AS. We constructed a proteoglycan (PG)-induced AS mouse model and administered IAA (50 mg/kg body weight) by intraperitoneal injection daily for 4 weeks. The effects of IAA on AS mice were evaluated by examining disease severity, intestinal barrier function, aryl hydrocarbon receptor (AhR) pathway, T-helper 17 (Th17)/T regulatory (Treg) balance, and inflammatory cytokine levels. The intestinal microbiota compositions were profiled through whole-genome sequencing. We observed that IAA decreased the incidence and severity of AS in mice, inhibited the production of pro-inflammatory cytokines (tumor necrosis factor α [TNF-α], interleukin [IL]-6, IL-17A, and IL-23), promoted the production of the anti-inflammatory cytokine IL-10, and reduced the ratios of pro-/anti- inflammatory cytokines. IAA ameliorated pathological changes in the ileum and improved intestinal mucosal barrier function. IAA also activated the AhR pathway, upregulated the transcription factor forehead box protein P3 (FoxP3) and increased Treg cells, and downregulated the transcription factors retinoic acid receptor–related orphan receptor gamma t (RORγt) and signal transducer and activator of transcription 3 (STAT3) and decreased Th17 cells. Furthermore, IAA altered the composition of the intestinal microbiota composition by increasing Bacteroides and decreasing Proteobacteria and Firmicutes, in addition to increasing the abundances of *Bifidobacterium pseudolongum* and *Mucispirillum schaedleri*. In conclusion, IAA exerted several protective effects against PG-induced AS in mice, which was mediated by the restoration of balance among the intestinal microbial community, activating the AhR pathway, and inhibiting inflammation. IAA might represent a novel therapeutic approach for AS.

## Introduction

Ankylosing spondylitis (AS), the best-known subtype of axial spondyloarthritis (SpA) (1), is a systemic, chronic, inflammatory autoimmune disease that primarily affects the sacroiliac joints, axial skeleton, and peripheral joints, eventually leading to spine and joint ankylosis (2). The estimated prevalence of AS is 1%–2% in western populations (3) and approximately 0.2%–0.54% among the ethnic Han Chinese population (4). AS commonly occurs in young adult males aged between 20 and 30 years, and approximately 90% of patients with AS develop symptoms before the age of 40 years (5). AS is characterized by inflammation of the axial skeleton, including inflammatory back pain, the destruction of joint structures, pathological new bone formation, and peripheral manifestations, such as peripheral arthritis, enthesitis, or dactylitis. Many patients with AS also have extra-articular manifestations, including acute anterior uveitis (AUU), psoriasis, inflammatory bowel disease (IBD), and osteoporosis. AS can limit an individual’s functional activities, seriously affecting quality of life, inhibiting the working capacities of young patients, endangering personal physical and mental health, and imposing a considerable burden on both the patient and society (6, 7).

Human leukocyte antigen-B27 (HLA-B27) plays an important role in the pathogenesis of AS, with greater than 90% of AS patients identified as HLA-B27 positive. However, only 5% of HLA-B27 carriers develop AS (8), indicating that HLA-B27 is not the sole genetic factor that determines the development of AS. Growing evidence indicates pivotal roles for the intestinal microbiota and the IL-23/IL-17 axis in the pathogenesis of AS (1, 9). The human gastrointestinal tract is colonized by trillions of microorganisms, most of which are commensal bacteria, which influence the health and immune responses of the host (10). Over the last decade, the development of novel sequencing technologies has provided increasing evidence that the composition of the intestinal microbiota is involved in the pathogenesis of various inflammatory and autoimmune diseases, including AS (9), IBD (11), AUU (12), rheumatoid arthritis (RA) (13), systemic lupus erythematosus (SLE) (14), multiple sclerosis (MS) (15), and psoriasis (16). Genome-wide association studies have indicated the existence of a significant relationship between the pathogenesis of AS and imbalances in the intestinal microbiota composition (17). Breban et al. observed disease-specific intestinal dysbiosis in patients with AS compared with healthy individuals, which positively correlated with disease activity in patients with a history of IBD (18). In a similar study, terminal ileum biopsies of biologic-naïve AS patients showed a strong microbial imbalance compared with healthy controls, with the increased abundance of five bacterial families and the decreased abundance of two bacterial families (19). This finding suggested that intestinal microbiota composition is an essential factor in the pathogenesis of AS. Animal models have also indicated that the intestinal microbiota composition plays an important role in the development of AS. HLA-B27 transgenic rats do not appear features of AS in a germ-free environment. However, when transferred to a conventional rat colony or after the introduction of common luminal bacteria, over 80% of HLA-B27 transgenic rats developed AS and diarrhea (20). These findings suggest that the intestinal microbiota population may represent a potential target for AS treatment.

Indole-3-acetic acid (IAA) is the most biologically active auxin, able to regulate the growth and development of plants by enhancing cell proliferation and antioxidant effects (21, 22). IAA has been detected not only in plants but also in mammals (23). In mammals, IAA is an important indole-derivative catabolized from dietary tryptophan by the intestinal microbiota; therefore, intestinal dysbiosis can influence IAA production (24). IAA has been detected and quantified among human metabolites obtained from a variety of body parts and fluids, including feces, urine, saliva, blood, and cerebrospinal fluid (25). Recent evidence suggest that IAA can scavenge free radicals, inhibit oxidative stress, and mitigate pro-inflammatory cytokine production (22, 26). In addition, as a ligand of aryl hydrocarbon receptor (AhR), IAA also can modulate intestinal homeostasis and mucosal immunity by activating the AhR pathway (24). AhR activation not only promotes intestinal barrier function and regulates the intestinal microbiota composition but also attenuates inflammatory responses by modulating intestinal immune cells that produce anti-inflammatory cytokines, such as interleukin (IL)-10 and IL-22, as well as decreasing the production of pro-inflammatory cytokines, such as tumor necrosis factor-α (TNF-α), IL-6, IL-17A, and IL-23 (27, 28). However, whether IAA can exert protective effects against AS by inhibiting the inflammatory response triggered by the intestinal microbiota remains unknown. Therefore, in this study, we constructed an animal model of AS, investigated the ability of IAA to activate the AhR pathway, and explored the effects of IAA against AS, with particular focus on the inflammatory response, intestinal barrier function, and intestinal microbiota disorder. To the best of our knowledge, this study represents the first report to evaluate the effects of IAA on the gut-bone axis in AS.

## Materials and Methods

### Construction of the Proteoglycan-Induced AS Mouse Model and Experimental Protocol

Eight-month-old female BALB/c retired breeder mice (body weight 24 ± 2 g) were purchased from Guangzhou Chase Reward Co. Ltd, China. The mice were bred in the animal facility of the Fifth Affiliated Hospital of Sun Yat-sen University under controlled conditions (12-h light/12-h dark; 22 ± 2°C). All mice were maintained in a specific pathogen–free environment and received sterile food and filtered water ad libitum. All experimental protocols were performed following approval from the Institutional Animal Care and Use Committee of the Fifth Affiliated Hospital of Sun Yat-sen University.

The proteoglycan (PG)-induced AS model has been described previously (29). The mice were immunized on days 0, 21, and 42 by intraperitoneal injections with 200 µl of an emulsifier containing 100 µg of cartilage PGs (Sigma-Aldrich, St. Louis, MO, USA) in 100 µl PBS and 100 µl complete Freund’s adjuvant (Sigma-Aldrich, St. Louis, MO, USA).

**Figure 1:**
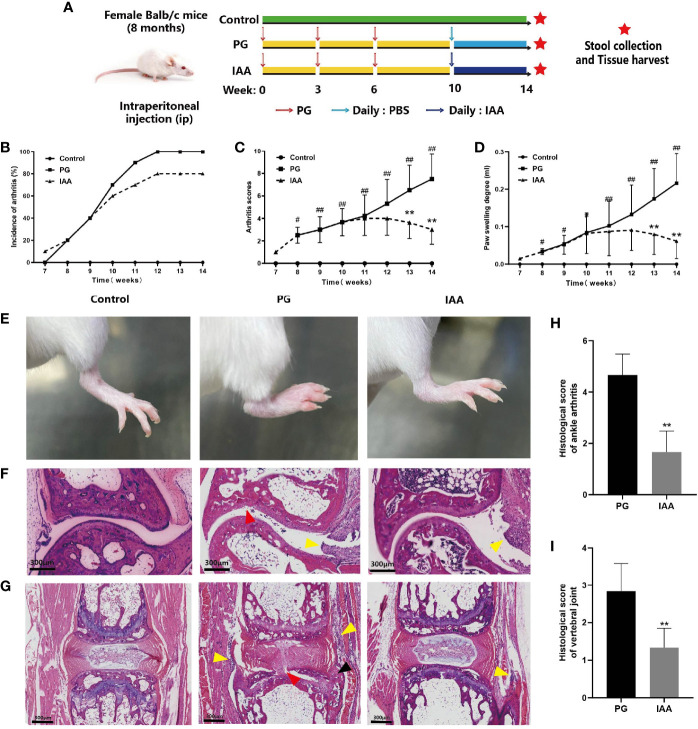
IAA attenuates disease progression and severity in PG-induced AS mice. **(A)** Schematic diagram for study design. Incidence of arthritis **(B)**, arthritis index score **(C)**, and degree of hind feet swelling **(D)** in three groups of mice (n = 10). **(E)** Representative pictures of the hind feet in three groups of mice at the end of week 14. **(F)** Representative pictures of ankle joint sections in three groups of mice (HE staining, scale bar = 300 µm). PG group was characterized by inflammatory cell infiltration in the synovium of the joint capsule (yellow arrow), cartilage degeneration, and bone erosion on the articular surface (red arrow). **(G)** Representative pictures of vertebral joint sections in three groups of mice (HE staining, scale bar = 300 µm). PG group was characterized by inflammatory cell infiltration in the ligaments around the intervertebral disc (yellow arrow), partial destruction of intervertebral disc structure (red arrow), and new chondrocytes (black arrow). **(H)** Histologic scores for ankle joint sections in the PG and IAA groups (n = 6). **(I)** Histologic scores of vertebral joint sections in the PG and IAA groups (n = 6). Data are expressed as the mean ± standard deviation (SD); ^#^ *p* < 0.05, ^##^ *p* < 0.01 vs. control; **p < 0.01 vs. PG alone. IAA, indole-3 acetic acid; PG, proteoglycan; AS, ankylosing spondylitis; HE, hematoxylin and eosin.

The mice were randomly and evenly divided into three groups (n = 10 per group): 1) control group containing 10 healthy mice; 2) AS model group (PG group), in which AS model mice received daily intraperitoneal injections of vehicle control (phosphate-buffered saline, PBS); and (3) IAA group, in which AS model mice received daily intraperitoneal injections of IAA (Sigma-Aldrich, St. Louis, MO, USA) at a dose of 50 mg/kg body weight. The IAA dosage used in the present study was based on previously published data (30, 31) and our preliminary experiment. This dosage was identified as safe for mice. IAA was completely dissolved in PBS (stock solution 10 mg/mL) by adding NaOH (1 N), followed adjusting the pH to 7.4 with 25% (v/v) HCl. IAA or vehicle treatment began in week 10, after PG induction, and continued for 4 weeks. The entire experiment lasted 14 weeks (**Figure 1A**).

### Clinical and Histological Assessments of Arthritis and Spondylitis

Arthritis severity was evaluated by two investigators blinded to the animal treatments by examining changes in the polyarthritis index and the volume of hind paw swelling. The polyarthritis index was scored weekly, starting after the third immunization (week 7) until euthanasia (week 14), using a standard visual scoring system (32): 0, no symptoms; 1, redness and swelling in one toe; 2, redness and swelling in more than one toe; 3, toe stiffness; and 4, deformity or ankle involvement. The accumulated maximum score, including all four paws for each mouse, was 16 points. Hind paw volumes were measured with a PV-200 volume meter (Chengdu Technology Market Co. Ltd., Sichuan, China) before and after immunization. Paw swelling degree was calculated as paw swelling at each time point (weekly during weeks 7–14) − paw swelling (baseline, week 0).

At the end of week 14, the mice were sacrificed. The hind paws and spine were dissected and fixed in buffered 10% formalin, followed by further decalcification in 10% Titriplex EDTA for 1 month and embedding in paraffin. Serial 5-µm sections were stained with hematoxylin and eosin (H&E), and images were acquired using a Pannoramic 250 (3DHISTECH, Budapest, Hungary). Stained sections were semi-quantitatively scored by two independent, blinded observers.

For arthritis score assessments, entire digital images were analyzed according to synovial inflammation, cartilage degradation, and bone erosion, as previously described (33). Synovial inflammation was scored using the following scale: 0, normal; 1, slight thickening of the lining layer, with some infiltrating cells in the sublining layer; 2, moderate thickening of the lining layer, with a moderate number of infiltrating cells in the sublining layer; and 3, severe inflammation with a massive immune cell infiltrate into the synovium. Cartilage degradation was scored using the following scale: 0, normal; 1, mild cartilage destruction; 2, evidence of cartilage destruction with synovial cell invasion; and 3, severe loss of cartilage. Bone erosion was scored using the following scale: 0, normal; 1, mild loss of cortical bone at a few sites; 2, moderate loss of cortical and trabecular bone; and 3, marked loss of bone at many sites. The scores for each criterion were added together to obtain an overall inflammation score for each sample, ranging from 0 to 9 points.

For the vertebral joint score assessment, entire digital images were analyzed according to the level of inflammation, as previously described (34). Inflammation was scored using the following scale: 1, enthesitis, inflammatory cell accumulation around the intervertebral disks (IVD) or infiltration of the annulus fibrosus; 2, <50% absorption/erosion of the IVD; 3, essentially complete resorption (> 50%) of the IVD; and 4, cartilaginous/bony ankylosis.

### Enzyme-Linked Immunosorbent Assay

At the end of week 14, the mice were anesthetized by exposure to 3% isoflurane. Approximately 1 mL of blood was obtained from each mouse *via* eyeball enucleation and allowed to clot at room temperature for 1 hour. Serum samples were separated by centrifuging at 2000 × *g* for 10 min at 4°C and stored at −80°C until analysis. IL-6, TNF-α, IL-17A, IL-23, and IL-10 levels were measured using a commercial enzyme-linked immunosorbent assay (ELISA) kit (Beyotime Biotechnology, Shanghai, China), following the manufacturer’s instructions.

### Assessment of Intestinal Inflammation

At the end of week 14, intestinal tissues were quickly removed following sacrifice. After irrigation with PBS, some intestinal tissue samples of each individual mouse were immediately stored at −80°C for future western blot analysis, whereas others were immediately fixed in buffered 10% formalin. Paraffin-embedded sections 4µm-thick were stained with H&E, and images were acquired using Pannoramic 250. Inflammation was evaluated in a blinded manner by two investigators using a previously described scoring system (35, 36). Entire digital images were analyzed based on three criteria: inflammatory cell infiltration, epithelial changes, and mucosal architecture. Inflammatory cell infiltration was scored as follows: 1, mild mucosal infiltration; 2, moderate mucosal and submucosal infiltration; and 3, marked mucosal, submucosal, and transmural infiltration. Epithelial changes were scored as follows: 1, mild hyperplasia (<25%); 2, moderate hyperplasia (25%–50%); and 3, marked hyperplasia (>50%). The mucosal architecture was scored as follows: 1, mild villous blunting (villous-to-crypt-length ratio of 2:1 to 3:1); 2, moderate villous blunting (villous-to-crypt-length ratio of 1:1 to 2:1); and 3, marked, villous atrophy, branched crypts. The scores for each criterion were combined to obtain an overall inflammation score for each sample, ranging from 0 to 9 points.

### Immunohistochemistry

Immunohistochemistry staining for zonula occludens-1 (ZO-1) and Occludin, types of tight junction proteins in the intestine, in 4µm-thick paraffin-embedded intestinal sections were performed as previously described (37). Briefly, tissue sections on slides were deparaffinized in xylene, rehydrated in graded alcohol and washed in PBS, immersed in 3% hydrogen peroxide in PBS for 15 min at room temperature to block endogenous peroxidase. We perform heat mediated antigen retrieval with Tris/EDTA buffer pH 9.0 (ab 93684, Abcam, Cambridge, UK). After washing with PBS buffer, the sections were treated with goat serum for 20 min at room temperature to block nonspecific binding. Subsequently, the sections were incubated overnight at 4°C with primary antibodies: rabbit monoclonal anti-ZO-1 (1:1000; ab221546, Abcam, Cambridge, UK) or rabbit monoclonal anti-occludin (1:200; ab216327, Abcam, Cambridge, UK), and then, incubated with horseradish peroxidase (HRP)-conjugated goat against rabbit IgG secondary antibody (1:1000; ab6721, Abcam, Cambridge, UK) for 30 min at room temperature. The immunocomplexes were visualized using diaminobenzidine (DAB), and the nuclei were counterstained with hematoxylin. The images were acquired using Pannoramic 250 and analyzed using ImageJ v1.46r software by measuring the optical density of ZO-1 and occludin proteins. The arithmetic mean of the quantification in five fields per section was determined.

### Western Blot Analysis

Total proteins were extracted from intestinal tissue by homogenization in radioimmunoprecipitation assay (RIPA) buffer. The bicinchoninic acid (BCA) protein assay kit (Beyotime Biotechnology, Shanghai, China) was used to determine protein concentrations. Equal amounts of protein (40 µg) were separated by 10% sodium dodecyl sulfate–polyacrylamide gel electrophoresis (SDS-PAGE), followed by transfer to a polyvinylidene difluoride (PVDF) membrane (Millipore, Boston, MA, USA) using a semidry transfer system (Bio-Rad, Hercules, CA, USA). The membrane was blocked for 1 h at room temperature with 5% bovine serum albumin (BSA) and incubated overnight at 4°C with the following primary antibodies: anti-zonula occludens-1 (ZO-1) (1:1000; ab96587, Abcam, Cambridge, UK), anti-occludin (1:1000; ab167161, Abcam, Cambridge, UK), anti-AhR (1:1000; ab28698, Abcam, Cambridge, UK), anti-FoxP3 (1:1000; ab75763, Abcam, Cambridge, UK), anti-STAT3 (1:1000; ab68153, Abcam, Cambridge, UK), and anti-RORγt (1:1000; 14-6988-82, Thermo Fisher Scientific, Carlsbad, MA, USA). Appropriate HRP-conjugated secondary antibodies (1:3000; ab6721 and 31470) were obtained from Abcam and Thermo Fisher Scientific and incubated with the membrane for 1 h at room temperature. Bands were visualized with an enhanced chemiluminescence (ECL) reagent (Beyotime Biotechnology, Shanghai, China) using the ChemiDoc™ XRS+ imaging system (Bio-Rad, Hercules, CA, USA). The ImageJ v1.46r software was used to measure the optical density of protein bands.

### Flow Cytometry

Lamina propria (LP) immune cells from the small intestine were isolated using mouse lamina propria dissociation kit (130-097-410, Miltenyi Biotec, Bergisch Gladbach, Germany) according to the manufacturer’s instruction. For intracellular analysis of cytokine expression, cells were stimulated in culture medium with cell stimulation cocktail plus protein transport inhibitors (00-4975-93, eBioscience, San Diego, CA, USA) in a cell incubator with 5% CO2 at 37°C for 6h to promote IL-17 and FoxP3 expression and prevent secretion. To discriminate live from dead cells, single-cell suspensions were incubated with Zombie Aqua™ Fixable Viability Kit (1:100; 423101, Biolegend, San Diego, CA, USA) for 30 minutes in the dark at room temperature. For T-helper 17 (Th17) cells, cells were surface stained on ice for 20 min in the dark with surface markers APC/Cyanine7 anti-mouse CD3ϵ (145-2C11, Biolegend, San Diego, CA, USA) and FITC anti-mouse CD4 (RM4-5, Biolegend), then fixed, permeabilized and intracellular stained for 25 min at room temperature with intracellular markers PE anti-mouse IL-17A (TC11-18H10.1, Biolegend). For T regulatory (Treg) cells, cells were surface stained on ice for 20 min in the dark with surface markers FITC anti-mouse CD4 (RM4-5, Biolegend) and APC anti-mouse CD25 (PC61, Biolegend), then fixed, permeabilized and intracellular stained for 30 min at room temperature with intracellular markers PE anti-mouse FoxP3 (MF-14, Biolegend). All appropriate isotype controls were used as negative controls. Cell samples were acquired using a CytoFlex flow cytometer (Beckman Coulter, Brea, CA, USA), and data were analyzed using FCS Express v7.12 (*De Novo* Software, Pasadena, CA, USA).

### Metagenomic Sequencing and Bioinformatic Analyses

Fresh stool samples were collected from mice at week 14, placed into sterile containers, and immediately frozen at −80°C until processing. Microbial DNA was extracted from the fecal material of each sample using the TIANamp Genomic DNA Kit DP328 (TIANGEN Co., Beijing, China), according to the manufacturer’s instructions. The DNA concentration was measured using the Qubit^®^ dsDNA HS Assay Kit in a Qubit^®^ 2.0 Fluorometer (Thermo Fisher Scientific, Carlsbad, MA, USA). Sequencing libraries were generated using NEBNext^®^ Ultra™ DNA Library Prep Kit for Illumina (New England Biolabs, Ipswich, MA, USA), following the manufacturer’s recommendations. DNA libraries were validated using an Agilent 2100 Bioanalyzer (Agilent Technologies, Santa Clara, CA, USA). Finally, DNA libraries were sequenced on an Illumina NovaSeq 6000 (Illumina Inc., San Diego, USA), and paired-end reads were generated.

The sequencing resulted in approximately 60 Gb raw reads, with an average number of 4,324,296,072 reads per sample, ranging from 4,053,396,509 to 5,110,089,364. The raw sequence reads underwent quality control, *de novo* assembly, and gene prediction using the MetaPhlAn3 (version 3.0) pipeline (38). The adaptor was detected and trimmed using Trim Galore (version 0.6.4; https://github.com/FelixKrueger/TrimGalore), and Fastp (39) was used to remove reads with more than 10% N, Q20 >20%, or shorter than 60 bp. Mouse genomic reads were filtered by mapping the metagenomic reads against the latest mouse genome reference (mm10) using Bowtie2 (version 2.3.5.1) (40). Taxonomic annotation was generated using MetaPhlAn3 with the v30 version of the database and default parameters. Functional profiling based on metagenomic sequences was performed using the HUMAnN3 pipeline (version 3.0.0) (41) with default parameters to obtain gene families (UniRef90, 201901) (42).

Sequences were subjected to alpha and beta diversity analyses using the Vegan package, and visualization was performed using the ggplot2 package in R statistics software (version, R 3.6.3). Differences in alpha diversity were calculated according to the richness indicated by observed species and the diversity indicated by the Shannon index. Beta diversity was evaluated using principal coordinate analysis (PCoA) based on Bray–Curtis distance matrices. Permutational multivariate analysis of variance (PERMANOVA) was used to evaluate the effects of grouping variables on sample differences using the Vegan package in R statistics software. Heat map and clustering analyses were performed using the Vegan package in R statistical software. Significant differences in the relative abundance of gut microbiota between the control, PG, and IAA groups were determined using linear discriminant analysis (LDA) effect size (LEfSe) analysis (43), based on the Kruskal–Wallis rank-sum test. Only LDA scores > 3 and p-values < 0.05 (Kruskal–Wallis test) were considered significantly enriched.

For metagenomic analysis, significant differentially abundant genes identified in Kyoto Encyclopedia of Genes and Genomes (KEGG) pathways between the three groups were identified using the Kruskal–Wallis rank-sum test with Benjamini–Hochberg false discovery rate (FDR) correction.

### Statistical Analysis

All data are presented as the mean ± standard deviation (SD) and were analyzed using GraphPad Prism 8.3 (GraphPad Software, California, USA). Significant differences between the three groups were determined by one-way analysis of variance (ANOVA), followed by Bonferroni’s multiple comparisons test. A *p*-value < 0.05 was considered significant.

## Results

### IAA Attenuates Disease Progression and Severity in PG-Induced AS Mice

To evaluate the effectiveness of IAA treatment on arthritis, we examined arthritis incidence and arthritis severity in mice from the three treatment groups. The experimental design is summarized in **Figure 1A**. Compared with control mice, mice from the PG group began to develop redness and swelling in the foot and toes starting at week 7, which gradually aggravated over time and showed significant redness and swelling at week 14. This effect was alleviated by IAA treatment, as the incidence of arthritis was reduced and the redness and swelling were relieved to varying degrees during weeks 10–14 among the mice in the IAA group (**Figure 1B**). The arthritis scores and hind paw swelling in the IAA-treated mice were significantly reduced compared with those in the PG group during weeks 13–14 (*p* < 0.05, **Figures 1C, D**). Representative pictures of the hind feet from the different groups are shown in **Figure 1E**.

The histological sections obtained from the ankle joint of mice in the control group showed normal histological structures, with no signs of inflammatory infiltration or cartilage destruction and a clear synovial space. The ankle joints from mice in the PG group exhibited marked signs of arthritis, characterized by synovial proliferation, inflammatory cell infiltration, cartilage degeneration, and bone erosion (**Figure 1F**). Similarly, we observed inflammatory cell infiltration around the IVD and the destruction of the disk structure in the vertebral joints of mice from the PG group (**Figure 1G**). These histological features of the ankle joint and the vertebral joints were significantly ameliorated in IAA-treated mice. The histological scores for the ankle joint and the vertebral joints in the IAA group were significantly decreased compared with those in the PG group (*p* < 0.05, **Figures 1H, I**). Overall, these findings demonstrated that IAA treatment could inhibit the development and severity of AS in mice.

### IAA Reduces the Expressions of Pro-Inflammatory Cytokines, Increases the Expression of an Anti-Inflammatory Cytokine, and Reduces the Ratios of Pro-/Anti-Inflammatory Cytokines in AS Mice

**Figure 2:**
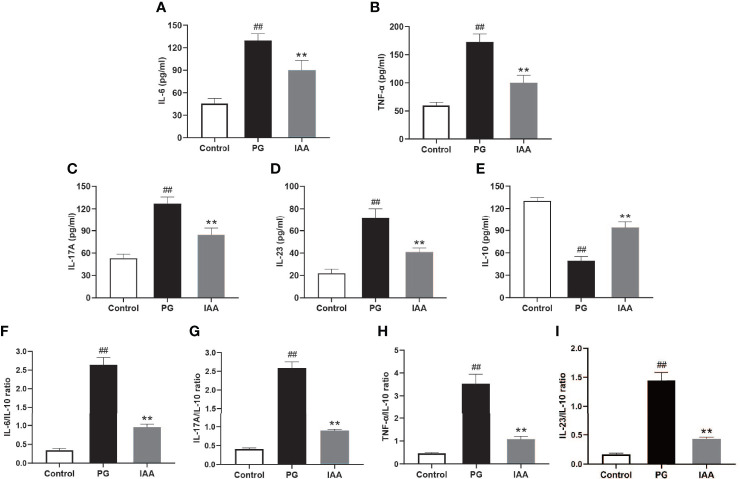
IAA inhibits pro-inflammatory cytokines expressions, elevates anti-inflammatory cytokine expression, and reduces the ratios of pro-/anti- inflammatory cytokines in AS mice. Ankylosing spondylitis (AS) mice were administered intraperitoneal injections of indole-3 acetic acid (IAA; 50 mg/kg/day) for 4 weeks. The serum levels of the pro-inflammatory cytokines interleukin (IL)-6, tumor necrosis factor (TNF)-α, IL-17A, and IL-23, and the anti-inflammatory cytokine IL-10 were detected by enzyme-linked immunosorbent assay (ELISA). **(A)** IL-6, **(B)** TNF-α, **(C)** IL-17A, **(D)** IL-23, **(E)** IL-10, **(F)** IL-6/IL-10, **(G)** IL-17A/IL-10, **(H)** TNF-α/IL-10, **(I)** IL-23/IL-10. (n = 6). Data are expressed as the mean ± standard deviation (SD); ^##^ *p* < 0.01 vs. control; **p < 0.01 vs. PG alone.

Inflammatory cytokines have been shown to play important roles in the pathogenesis of chronic inflammatory diseases and represent important therapeutic targets in human and animal models of these diseases (44). To assess the effects of IAA on systemic inflammatory cytokines, the circulating levels of pro-inflammatory and anti-inflammatory cytokines were examined. The serum levels of pro-inflammatory cytokines (TNF-α, IL-6, IL-17A, and IL-23) were significantly increased in the PG group compared with those in the control group, whereas IAA treatment significantly reduced the levels of TNF-α, IL-6, IL-17A, and IL-23 (*p* < 0.05, **Figures 2A–D**). By contrast, the serum level of the anti-inflammatory cytokine IL-10 was significantly decreased in the PG group compared with that in the control group, and IAA treatment significantly increased the level of IL-10 (*p* < 0.05, **Figures 2E**). Meanwhile, the ratios of pro-/anti- inflammatory cytokines (TNF-α/IL-10, IL-6/IL-10, IL-17A/IL-10, IL-23/IL-10) were significantly increased in the PG group compared with those in the control group, whereas IAA treatment significantly reduced the ratios of TNF-α/IL-10, IL-6/IL-10, IL-17A/IL-10, and IL-23/IL-10 (*p* < 0.05, **Figures 2F–I**). These results demonstrated that IAA could reduce the levels of pro-inflammatory cytokines, increase the level of an anti-inflammatory cytokine, and reduce the ratios of pro-/anti- inflammatory cytokines.

### IAA Ameliorates the Pathological Morphology of Ileum Tissue and Improves Intestinal Mucosal Barrier Function in AS Mice

**Figure 3:**
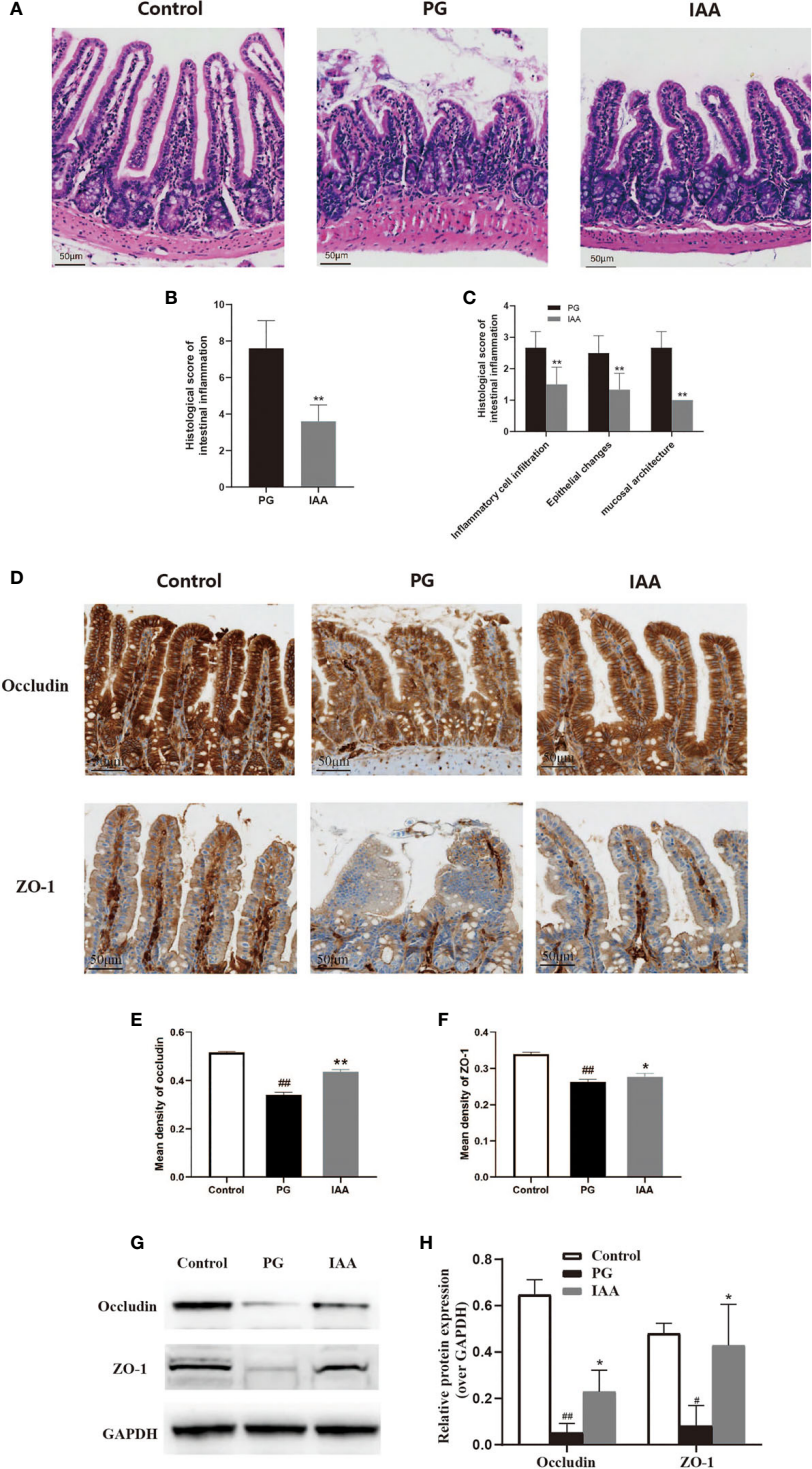
IAA ameliorates the pathological morphology of ileum tissue and improves intestinal mucosal barrier function in AS mice. **(A)** Representative pictures of ileum sections from three groups of mice at the end of week 14. (HE staining, scale bar = 50 µm). **(B)** Histologic scores for intestinal inflammation in the proteoglycan (PG) and indole-3 acetic acid (IAA) group (n = 6). **(C)** Histologic score of intestinal inflammation was divided into three categories: inflammatory cell infiltrates, epithelial changes, and mucosal architecture (n = 6). **(D)** The proteins of ZO-1 and occludin in ileum tissue from three groups of mice were detected by immunohistochemistry, as shown in representative pictures from one of three independent experiments (scale bar = 50 µm). **(E)** ZO-1 and **(F)** occludin staining intensities were quantified as the mean optical density (n = 6). **(G)** The tight junction protein (ZO-1 and occludin) levels in ileum tissue from three groups of mice were analyzed by western blot analysis, as shown in representative blots from one of three independent experiments. **(H)** Relative protein expression of ZO-1 and occludin using densitometric analysis, with GAPDH as a loading control (n = 3). Data are expressed as the mean ± standard deviation (SD); ^#^ *p* < 0.05, ^##^ *p* < 0.01 vs. control; *p < 0.05, **p < 0.01 vs. PG alone. HE, hematoxylin and eosin; ZO-1, zonula occludens-1; GAPDH, glyceraldehyde 3-phosphate dehydrogenase.

Intestinal inflammation is common among patients with AS and is thought to serve as a risk factor for AS development (45, 46). Thus, we investigated whether pathological alterations were observed in the intestinal mucosa of AS mice during AS development. We analyzed pathological sections of ileum tissue stained with H&E (**Figure 3A**). Histological analysis showed normal histological morphology in the control group, with intact epithelium, normal mucosal architecture, and no infiltration of inflammatory cells. Among the AS mice in the PG group, marked levels of epithelial hyperplasia, villous blunting, and inflammatory cell infiltration could be observed. IAA treatment significantly mitigated these pathological alterations, and the ileum samples from the IAA-treated group were characterized by mild levels of epithelial hyperplasia, villous blunting, and inflammatory cell infiltration. The histopathological intestinal inflammation score for the IAA group was significantly decreased compared with that for the PG group (*p* < 0.05, **Figures 3B, C**). Overall, IAA appeared to ameliorate pathological morphology in ileum tissue.

ZO-1 and occludin are important tight junction proteins, which are involved in intestinal leakage (47).. The decreased expression of these proteins increases intestinal permeability, which has been associated with AS pathogenesis (48). To examine whether IAA could protect the intestinal mucosal barrier function in AS mice, immunohistochemistry and western blot analysis were used to detect the expression of tight junction proteins in the ileum. The protein levels of ZO-1 and occludin were significantly decreased in the PG group compared with those in the control group. IAA treatment could partly restore ZO-1 and occludin protein levels (*p* < 0.05, **Figures 3D–H**), indicating that IAA could partially protect against PG-induced intestinal mucosal barrier dysfunction by enhancing intestinal tight junction expression and restoring intestinal integrity.

### IAA Activates the AhR Pathway and Regulates Th17/Treg Balance in AS Mice

**Figure 4:**
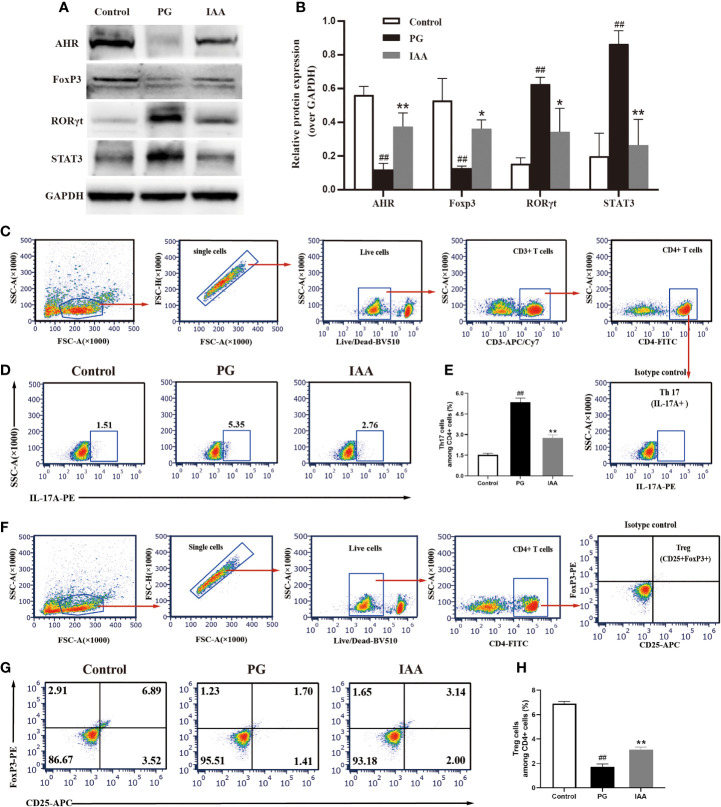
IAA activates AhR signaling pathway and restores Th17/Treg balance in AS mice. **(A)** The protein levels of aryl hydrocarbon receptor (AhR) and Th17/Treg-related transcription factors, such as forkhead box protein P3 (FoxP3), signal transducer and activator of transcription 3 (STAT3), and retinoic acid receptor–related orphan receptor gamma (RORγt) in ileum tissue from three groups of mice were analyzed by western blot analysis, as shown in representative blots from one of three independent experiments. **(B)** Relative protein expression of AhR, FoxP3, STAT3, and RORγt using densitometric analysis, with glyceraldehyde 3-phosphate dehydrogenase (GAPDH) as a loading control (n=3). **(C-H)** Flow cytometric analysis of the frequencies of Th17 and Treg cells in ileum lamina propria from three groups of mice. **(C)** Gating strategy used for analysis of Th17 cells. Arrows indicate that Th17 cells were sequentially gated from single cells, live cells, CD3^+^ cells and CD4^+^ cells. Gated CD4^+^ cells were analyzed for expression of IL-17A. Representative plots of IL-17A expression **(D)** and percentage of IL-17A^+^ cells **(E)** among CD4^+^ T cells (n=6). **(F)** Gating strategy used for analysis of Treg cells. Arrows indicate that Treg cells were sequentially gated from single cells, live cells, and CD4^+^ cells. Gated CD4^+^ cells were analyzed for expression of CD25 and Foxp3. Representative plots of CD25 and Foxp3 expression **(G)** and percentage of CD25+Foxp3^+^ T cells **(H)** among CD4+ T cells (n = 6). Data are expressed as the mean ± standard deviation (SD); ^##^ *p* < 0.01 vs. control; *p < 0.05, **p < 0.01 vs. PG alone. IAA, indole-3 acetic acid; Th17, T-helper 17; Treg, T regulatory; AS, ankylosing spondylitis.

AhR is a ligand-activated transcription factor that plays critical roles in various autoimmune diseases, including the regulation of Th17/Treg response (49, 50). An imbalance in the Th17/Treg response and the release of associated cytokines are thought to be involved in the pathogenesis of AS. The transcription factor FoxP3 is essential for Treg cell development and function (51). STAT3 and RORγt are important transcription factors involved in Th17 cell differentiation (52, 53). Th17/Treg balance is regulated by these important transcription factors. To further investigate the anti-inflammatory effects of IAA through the regulation of AhR activation and Th17/Treg balance, we first used western blot analysis to detect the expression of AhR and Th17/Treg-related transcription factors, such as RORγt, STAT3, and FoxP3 in the ileum. The protein levels of AhR and FoxP3 were significantly downregulated, and RORγt and STAT3 were significantly upregulated in the PG group compared with the control group. After IAA treatment, the protein levels of AhR and FoxP3 were significantly upregulated, and RORγt and STAT3 were significantly downregulated, in contrast to the PG group (*p* < 0.05, **Figures 4A, B**). These findings indicated that IAA could activate the AhR pathway and regulate Th17/Treg-related transcription factor activity. Due to the increased expression of Foxp3 and decreased expression of RORγt and STAT3, we next investigated whether IAA could regulate Th17/Treg balance by increasing Treg cell numbers and decreases Th17 cell numbers in AS model mice. We used flow cytometry to measure the percentages of Treg cells (CD4^+^CD25^+^Foxp3^+^) and Th17 cells (CD3^+^CD4^+^IL-17^+^) among CD4^+^ T cells in the lamina propria of ileum. The percentage of Th17 cells was increased and that of Treg cells was decreased in the PG group compared with those in the control group. As expected, IAA treatment markedly decreased the percentage of Th17 cells and increased that of Treg cells among CD4^+^ T cells in the ileal lamina propria of AS model mice (p < 0.05, **Figures 4C–H**). Taken together, these findings indicated that IAA could activate the AhR pathway and regulate Th17/Treg balance.

### IAA Modulates the Diversity and Composition of Intestinal Microbiota in AS Mice

**Figure 5:**
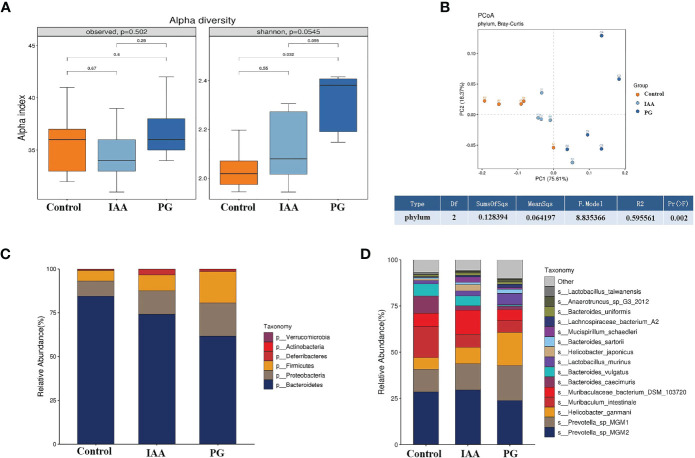
IAA modulates the diversity and composition of the intestinal microbiota in AS mice. **(A)** Alpha diversity in three groups was assessed by observed species and Shannon index. **(B)** Beta diversity at the phylum level in three groups was assessed by principal coordinate analysis (PCoA) of Bray–Curtis dissimilarity paired with permutational multivariate analysis of variance (PERMANOVA). Relative abundance analyses of intestinal microbiota composition at the phylum **(C)** and species **(D)** levels in three groups. (n = 3). IAA, indole-3 acetic acid; AS, ankylosing spondylitis.

We performed metagenomic sequencing on fecal samples obtained from the three groups to investigate changes in the intestinal microbiota compositions. We analyzed the diversity of the intestinal microbiota examined whether diversity was associated with AS. Alpha diversity was measured according to the observed species and the Shannon index, which showed no significant differences between the three groups (*p* = 0.502 and 0.0545, respectively, **Figure 5A**). However, comparative analysis revealed that the Shannon index value increased significantly in the PG group compared with the control group (*p* < 0.05, **Figure 5A**). To identify discrepancies between the intestinal microbiota compositions among the three groups, we assessed overall differences in beta diversity using a PCoA of the Bray–Curtis dissimilarity analysis, paired with PERMANOVA. The results showed that the microbiota community composition of the PG group was distinctly different from that of the control group (PERMANOVA R^2^ = 0.596, *p* = 0.002, **Figure 5B**). These findings showed that the richness and diversity of the intestinal microbiota composition found in AS mice were significantly different from those in control mice, suggesting that AS mice were characterized by disorder among the intestinal microbiota.

Subsequently, we analyzed the composition of the intestinal microbiota and detected whether changes in composition were associated with AS development. Taxon analysis showed differential abundance in the intestinal microbial compositions of the control, IAA, and PG groups. At the phylum level, Bacteroidetes, Proteobacteria, and Firmicutes were the three most dominant taxa (relative abundance >5%), representing greater than 96.5% of the total sequences across all three groups (**Figure 5C**). The relative abundance of Bacteroidetes (61.57%) decreased, and the relative abundances of Proteobacteria (18.97%) and Firmicutes (17.89%) increased in the PG group compared with the control group (84.41%, 8.62%, and 5.95%, respectively). IAA treatment increased the abundance of Bacteroides to 74.04% and decreased the abundances of Proteobacteria to 13.51% and Firmicutes to 9.05%. Moreover, the ratios of Bacteroides/Firmicutes and Bacteroides/Proteobacteria were 3.44 and 3.25 in the PG group, respectively, compared with 14.18 and 9.79 in the control group. IAA treatment increased the ratio of Bacteroides/Firmicutes to 8.19 and the ratio of Bacteroides/Proteobacteria to 5.48.

**Figure 6:**
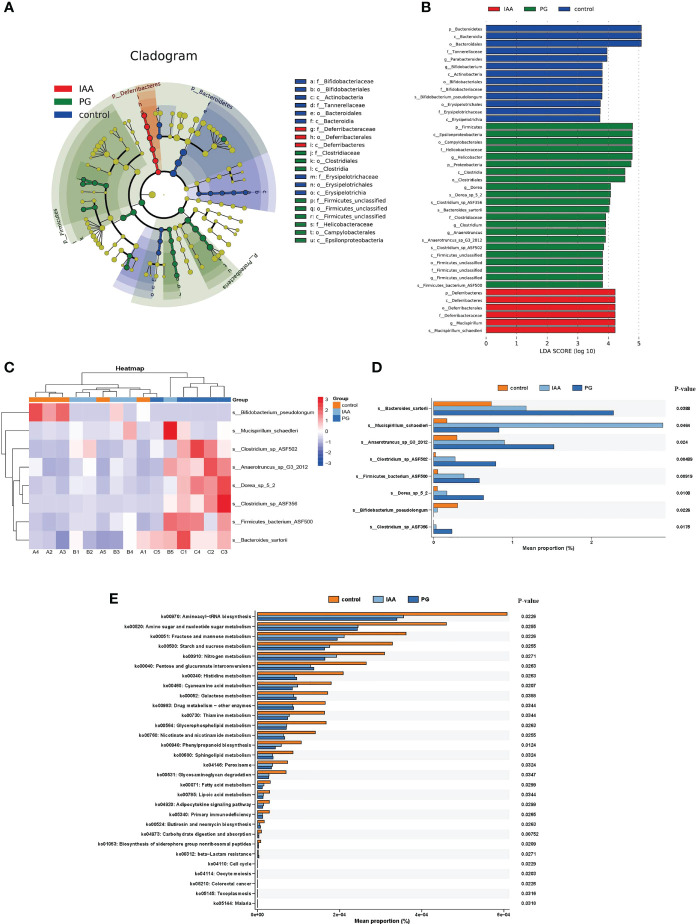
Comparison of the taxa and functional profiles of intestinal microbiota in three groups. The linear discriminant analysis effect size (LEfSe) analysis of the intestinal microbiota at the species level in three groups was presented on the cladogram **(A)** and bar plot **(B)** with linear discriminant analysis (LDA) score ≥ 3 and *p* < 0.05. **(C)** Heat map of 8 significantly different taxon abundances at the species level in three groups. **(D)** Box plot generated by rank-sum test (Kruskal–Wallis test) of relative abundance among the intestinal microbiota at the species level in three groups. **(E)** The differential analysis of functional predictions in three groups was performed based on level 3 of the Kyoto Encyclopedia of Genes and Genomes (KEGG) functional categories.

At the species level, we also found differences in the intestinal microbial community compositions among the three groups (**Figure 5D**). To identify specific individual bacterial taxa, which were differentially abundant across the three groups, we performed LEfSe analysis, using LDA ≥ 3 and p < 0.05 as the criteria to determine whether differences existed in these biomarkers between the three groups. A total of 41 differentially abundant bacterial taxa were identified across the three groups, including 22 bacterial taxa that were significantly more prominent in the PG group, 13 bacterial taxa that were significantly more prominent in the control group, and 6 bacterial taxa that were highly enriched in the IAA group (**Figures 6A, B**). Hierarchical cluster analysis, visualized using a heat map, showed 8 abundant taxa that were significantly different between groups at the species level (**Figure 6C**). The relative abundances of *Bacteroides sartorii, Anaerotruncus* sp. G3 (2012)*, Clostridium* sp. ASF502*, Firmicutes* bacterium ASF500*, Dorea* sp. 5-2, and *Clostridium* sp. ASF356 were significantly higher in the PG group, and the relative abundance of *Bifidobacterium pseudolongum* was significantly lower compared with those in the control group. Interestingly, IAA treatment significantly reversed these changes in species abundance associated with AS in the PG group. In addition, the abundance of *Mucispirillum schaedleri* was significantly higher in the IAA group than in the other two groups (**Figure 6D**, Kruskal–Wallis, *p* < 0.05).

To investigate the functional profiles of the intestinal microbiota compositions among the three groups, we performed a metagenomic analysis of our samples using KEGG pathway analysis in the HUMAnN3 pipeline. In total, we identified 63 KEGG pathways that were differentially abundant in the three groups based on the results of the Kruskal–Wallis test (Benjamini–Hochberg FDR correction). The significantly enriched KEGG pathways identified among the intestinal microbiota of AS mice were primarily distributed in cell growth and death pathways (e.g., oocyte meiosis and cell cycle), drug resistance: antimicrobial (e.g., beta-Lactam resistance), and infectious disease: parasitic (e.g., malaria and toxoplasmosis), indicating that these pathways were significantly disrupted and may potentially be involved in the pathogenesis of AS. After IAA treatment, enriched pathways were primarily distributed in genetic information processing (e.g., sorting and degradation), organismal systems (e.g., ‘development and regeneration’ and ‘excretory system’), environmental information processing (e.g., signal transduction), and metabolism, which included metabolism of cofactors and vitamins (e.g., retinol metabolism), xenobiotic biodegradation and metabolism (e.g., ‘nitrotoluene degradation’, ‘caprolactam degradation’, and ‘ethylbenzene degradation’), metabolism of terpenoids and polyketides (e.g., ‘Geraniol degradation’, ‘Limonene’, and ‘pinene degradation’), and biosynthesis of other secondary metabolites (e.g., penicillin and cephalosporin biosynthesis) (**Figure 6E**). The enrichment pathways in the IAA group were similar to those in the control group, suggesting that IAA treatment was able to recover the function of the intestinal microbiota in AS mice.

## Discussion

Although the etiology and pathogenesis of AS remain unclear, growing evidence has revealed that intestinal dysbiosis is involved in the occurrence and development of AS (17–19). Therefore, the restoration of intestinal microbiota balance is likely to contribute to AS treatment. Intestinal dysbiosis results in increased intestinal permeability, abnormal immune activation, and inflammation (54–56). Tryptophan metabolites are important signaling molecules within microbial communities, in addition to mediating host-microbial crosstalk, and play crucial roles in enhancing the intestinal epithelial barrier function, regulating the immune and inflammatory responses, and modulating the intestinal microbial composition, which contributes to intestinal and systemic homeostasis (24). Increasing evidence indicates that the complex commensal bacteria that inhabit the mammalian gastrointestinal tract have versatile impacts on intestinal tryptophan availability, thus being considered collectively as a driving force affecting the tryptophan metabolism in the gut (57). Indole and its derivatives, such as indole, IAA, indole-3- propionic acid (IPA), indoleacrylic acid (IA), indole-3-lactic acid (ILA), and indole-3-aldehyde (IAld), are important gut microbiota-derived tryptophan metabolites and have been reported as specific ligands of AhR, which can activate the AhR signaling pathway (27). AhR activation contributes to regulate intestinal immunity, inflammation and maintain intestinal homeostasis (58). Indole can enhance epithelial barrier function and promote intestinal mucosal homeostasis (59). IPA has antineoplastic features in breast cancer (60) and effect on astrocytes to limit central nervous system (CNS) inflammation (61), as well as inhibit colonic inflammation induced by dextran sodium sulfate (DSS) in mice (62). IA can promote intestinal epithelial barrier function and mitigate inflammatory responses (63). IAld can regulate IL-22-dependent balanced mucosal response, which provides antifungal resistance and mucosal protection from inflammation (64). ILA have been found to reprogram intraepithelial CD4^+^ T helper cells into Treg cells and inhibit Th17 cells polarization *in vitro*, which plays an important role in autoimmune and inflammatory diseases (65). IAA can also inhibit the inflammatory response and maintain intestinal homeostasis (26). IAA is formed from indole-3-acetamide, which is converted from tryptophan by tryptophan 2-monooxygenase of bacteria, such as Clostridium, Bacteroides, and Bifidobacterium (57). Recent studies have shown that the level of IAA is decreased in DSS-induced colitis mouse and the strains capable of metabolizing tryptophan or AHR agonist can inhibit intestinal inflammation (66). In rheumatoid arthritis study, the level of IAA is also lower in collagen-induced arthritis (CIA) rat and human umbilical mesenchymal stem cells treatment can increase the level of IAA and reduce disease severity of arthritis (50). Similarly, the level of IAA is also decreased in human subjects with IBD and SpA (67). Therefore, we focused on the underlying mechanisms through which IAA protects against AS development. In the present study, we clarified the protective effects and mechanism associated with IAA treatment, which mitigated the severity of PG-induced AS in model mice, characterized by the amelioration of inflammation, intestinal barrier breakdown, and intestinal microbiota disorder, in addition to the activation of the AhR pathway and the regulation of numerous transcription factor activities, which indicate that IAA is a potential molecule that might be developed for use in the treatment of AS. Our findings provided *in vivo* evidence, for the first time, to support the protective role of IAA against AS development.

In our study, we found that the AS model mice displayed marked clinical and histological features of arthritis, with significantly higher arthritis scores and histological scores compared with control mice. Interestingly, IAA treatment was able to significantly alleviate the inflammatory response and reduce joint destruction in AS model mice, suggesting that IAA treatment was able to slow AS disease progression and relieve disease severity in a model mouse.

AS is a complex chronic inflammatory state that is closely related to the expression of pro-inflammatory cytokines, such as IL-6, TNF-α, IL-17A, and IL-23, which are significantly upregulated in the peripheral blood of patients with AS (68). These pro‐inflammatory cytokines have been shown to play important roles in the pathogenesis of AS and have been identified as biomarkers of disease severity (69). In recent years, multiple clinical trials have examined the effects of IL-6, TNF-α, IL-17A, and IL-23 inhibitors in patients with AS (70–74), which showed that blocking these pro‐inflammatory cytokines was able to partially alleviate inflammatory symptoms and reduce disease severity. Among these, the IL-17A and TNF-α inhibitors appear to be the most effective biologic drugs for the treatment of AS. Whether IAA is able to inhibit these pro‐inflammatory cytokines is worthy of further study. Bansal et al. found that tryptophan-derived bacterial metabolites were able to attenuate inflammatory indicators in epithelial cells, characterized by the decreased expression of the pro-inflammatory cytokine TNF-α and the pro-inflammatory chemokine IL-8, in addition to the increased expression of the anti-inflammatory cytokine IL-10 (59). Krishnan et al. found that IAA significantly decreased the mRNA levels of TNF-α, IL-1β, and monocyte chemoattractant protein (MCP)-1 in macrophages exposed to palmitate and lipopolysaccharide (LPS) (26). Similarly, Ji et al. found that IAA attenuated the inflammatory response in the liver in high-fat diet-fed mice, as indicated by reduced mRNA levels of inflammatory cytokines, including MCP-1 and TNF-α (31). These findings were similar to our results. We found that AS mice displayed increased circulating levels of pro-inflammatory cytokines, such as IL-6, TNF-α, IL-17A, and IL- 23 and a decrease in the circulating level of anti-inflammatory cytokine IL-10, as well as increased ratios of pro-/anti- inflammatory cytokines, such as TNF-α/IL-10, IL-6/IL-10, IL-17A/IL-10, and IL-23/IL-10. However, IAA was able to significantly decrease the levels of pro-inflammatory cytokines, increase the level of an anti-inflammatory cytokine, and decrease the ratios of pro-/anti- inflammatory cytokines in AS mice. These results illustrated that IAA could slow AS progress and relieve disease severity by affecting the levels of various inflammatory cytokines.

The intestinal mucosal barrier plays an important role in maintaining intestinal homeostasis by preventing antigenic molecules and luminal microbes from penetrating the intestinal mucosa (75). Dysfunction of the intestinal mucosal barrier can increase intestinal permeability and trigger an immunological response, contributing to the development of various autoimmune diseases (76). Commonly, AS patients suffered from intestinal dysbiosis, resulting in the disruption of the intestinal mucosal barrier, increased intestinal permeability, and intestinal inflammation. Therefore, we further investigated whether the anti-inflammatory effects of IAA against AS were associated with any effects on intestinal mucosal barrier function. Tight junction proteins play critical roles in maintaining the integrity of the intestinal mucosal barrier. ZO-1 and Occludin are the most important tight junction proteins responsible for intestinal mucosal barrier function (77). A recent study found that enhanced IAA levels were associated with improved intestinal mucosal barrier integrity (78). IAA likely improves intestinal mucosal barrier function by activating the intestinal AhR pathway, which enhances the abundance of ZO-1 and occludin proteins (78). In the present study, we found that the protein expression levels of occludin and ZO-1 proteins significantly decreased in AS mice. However, IAA was able to partially restore intestinal mucosal barrier function by enhancing the expression of occludin and ZO-1 proteins. Moreover, we also identified the presence of subclinical intestinal inflammation in AS mice. IAA significantly increased the villus height and decreased epithelial hyperplasia and inflammatory cell infiltration in the ileum. These histomorphology parameters are considered indicators of intestinal health, with a healthy ileum mucosa characterized by long villi with high villus/crypt ratios (79). Therefore, our findings indicated that IAA could inhibit intestinal inflammation and promote intestinal health.

The intestinal mucosal barrier function is closely associated with the AhR pathway. AhR is a ligand-activated transcription factor that may serve as a receptor for multiple physiological ligands and is involved in the regulation of the mucosal immune response, inflammation, and intestinal homeostasis (27). AhR is expressed by various intestinal immune cells and affects their differentiation and function (80). In the context of inflammation, the AhR pathway can be activated to promote the transdifferentiation of Th17 cells into Treg cells, which can consequently amplify the anti-inflammatory effects of IL-10 and attenuate the pro-inflammatory effects of IL-17A, eventually suppressing inflammatory responses (81). IAA, an important tryptophan-derived bacterial metabolite, is a specific ligand for AhR and represents the dominant AhR agonist, able to activate the AhR pathway (24). Our present study showed that mucosal barrier destruction resulted in a significant decrease in AhR protein expression in the PG group, whereas IAA treatment was able to activate the AhR pathway, increasing AhR protein expression.

In the past few years, the IL-23/IL-17 axis has been considered to be a crucial contributor to the pathogenesis of AS (82). IL-23 is produced in large amounts by all antigen-presenting cells, such as dendritic cells, monocytes, and macrophages, and plays an important role in inducing IL-17A expression (83). IL-17A is primarily produced by Th17 cells under the control of IL-23 (84). The transcription factors STAT3 and RORγt are important transcription factors, which induce Th17 cells differentiation to produce IL-17A and enhance the expression of other pro-inflammatory cytokines, such as IL-6, IL-23, interferon-γ and TNF-α (53, 85–87). The transcription factor FoxP3 plays an essential role in the development and function of Treg cells, which regulate the release of anti-inflammatory cytokines, such as IL-10 (51). Previous studies have reported that RORγt or STAT3 deficient mice are resistant to autoimmune diseases (52, 88), and mice treated with FoxP3 displayed enhance Treg function and the alleviation of autoimmune diseases, such as IBD and experimental arthritis (89, 90). Similarly, an increasing number of studies have verified that the imbalance of the Th17/Treg cells with pro-/anti-inflammatory cytokines production is linked to the development and progression of AS (91, 92). Our present study showed that the protein expression levels of RORγt and STAT3 and the percentage of Th17 cells were significantly increased, whereas FoxP3 and Treg cells were significantly decreased in the PG group. The activation of the AhR pathway by IAA treatment significantly downregulated the expression of RORγt and STAT3 proteins and significantly upregulated the expression of FoxP3 protein, as well as decreased Th17 cell numbers and increased Treg cell numbers, which further inhibited the release of pro‐inflammatory cytokines, including TNF-α, IL-6, IL-17A, and IL-23, and promoted the release of the anti-inflammatory cytokine IL-10. These results illustrated that IAA treatment inhibited AS development by activating the AhR pathway, regulating the activity of numerous transcription factors, restoring Th17/Treg balance, and further inhibiting the IL-23/IL-17 inflammatory axis.

Intestinal dysbiosis, which refers to an imbalance in the intestinal microbiota population, disrupts the intestinal barrier and increases intestinal permeability, which is associated with several autoimmune diseases, such as IBD and AS (76). Growing evidence has revealed the involvement of the gastrointestinal tract in the development of AS. A strong association exists between IBD and AS, which have a high degree of co‐familiality (93). Ciccia et al. demonstrated that dysbiosis in the terminal ileum was associated with gut inflammation in AS patients (94).. A meta-analysis study that included a large population showed that the incidence of IBD in AS patients was 5.3-fold higher than that in healthy controls, and up to 13% of IBD patients developed AS (95). In a similar study, approximately 50% of AS patients had subclinical gut inflammation. Chronic gut inflammation was identified as a risk factor for active SpA and was associated with an increased risk of developing Crohn’s disease (45, 46). Furthermore, differences in the composition of the intestinal microbiota were observed between AS patients and healthy individuals, with evidence of dysbiosis in AS patients (19, 96). IAA treatment was able to improve intestinal mucosal barrier function and alleviate the inflammatory response, but the underlying mechanisms underlying the effects of IAA on the intestinal microbiota composition remain unclear. We, therefore, investigated the potential beneficial effects of IAA treatment on intestinal microbiota regulation.

Metagenomic sequencing and bioinformatic analyses were used to analyze the changes in the intestinal microbiota compositions among the three groups. Alpha diversity analysis revealed increased microbial diversity (Shannon index) in the AS group compared with the control samples, which was consistent with a previous report (19). Beta diversity analysis showed that the community compositional structures were significantly different between the three groups, which supported the previous findings (18) and suggested the presence of an imbalance in the intestinal microbiota composition in AS mice.

Bacteroidetes, Firmicutes, Proteobacteria, and Actinobacteria are the four dominant taxa found in both AS patients and healthy controls (17). The Firmicutes/Bacteroidetes ratio can be used as a useful indicator for evaluating the balance of the intestinal microbiota population (97). An increased abundance of Firmicutes and an increase in the Firmicutes/Bacteroidetes ratio were reported for AS patients in a previous study (18). The abundance of Actinobacteria was also found to be significantly increased among AS patients compared with healthy controls (17). In addition, an increase in Firmicutes combined with a decrease in Bacteroidetes may lead to a switch of the immune response toward an inflammatory profile involving the activation of Th17 cells (98). In our study, we found the abundance of Firmicutes, Proteobacteria, and Actinobacteria were significantly increased, and the abundance of Bacteroides was significantly decreased in AS mice compared with control mice. IAA treatment was able to reverse these changes associated with intestinal microbiota dysbiosis. Therefore, IAA was able to partially restore the intestinal microbiota balance and maintain its stability.

At the species level, LEfSe analyses also revealed significant differences in the intestinal microbial community between these three groups. In our study, species *Bacteroides sartorii, Anaerotruncus* sp. G3 (2012), *Clostridium* sp. ASF502*, Firmicutes* bacterium ASF500*, Dorea* sp. 5-2, and *Clostridium* sp. ASF356 were greatly more abundant in the PG group compared with the control group but decreased after IAA treatment. The specific mechanism for pro-inflammatory activity of these species remains unclear and requires further investigation. The genus *Bifidobacterium* includes important probiotic species that are necessary for intestinal microbial homeostasis and can reduce inflammation by inducing the production of immunosuppressive Treg cells (99). *Bifidobacterium pseudolongum*, a member of the genus *Bifidobacterium*, is a key commensal intestinal bacterial species with immunomodulatory capacity (100) and is associated with positive health effects, including increased gut barrier integrity and reduced inflammation (101). We found that the abundance of *Bifidobacterium pseudolongum* was significantly decreased in the PG group compared with the control group, and IAA treatment could increase the abundance. *Mucispirillum schaedleri*, a member of the phylum Deferribacteres, is typically found at low levels among the intestinal microbiota of mammals and might protect mice against colitis (102). IAA treatment also significantly increased the abundance of *Mucispirillum schaedleri*. Thus, IAA increased the abundance of beneficial bacteria to maintain the stability of the intestinal microbiota composition, which was beneficial to overall host health. Therefore, the effects of IAA treatment on improved intestinal mucosal barrier function and alleviated inflammatory responses may also be mediated by the regulation of the intestinal microbiota composition, in addition to the activation of the AhR pathway.

Studies have reporting contrasting reports regarding the intestinal microbiota composition of AS mice, and no conclusive findings identifying AS-specific intestinal microbiomes have been published. Differences in the outcomes of various studies may be due to differences in the genetic background, infection status, intervention, or housing conditions of the animals being studied, which can all influence the composition of the intestinal microbiota (103). By considering these factors, future studies with better study designs and carefully controlled experimental conditions can further explore the definition of an AS-specific intestinal microbiome (104).

The metagenomic analysis of KEGG pathways identified differentially abundant pathways among the three groups. The PG group was characterized by the increased representation of pathways associated with cell growth and death, drug resistance: antimicrobial, and infectious disease, suggesting a more vigorous inflammatory state. IAA treatment was able to reduce the enrichment of these pathways, inhibiting the inflammatory response and improving the therapeutic effect. The enriched pathways identified in the IAA group were primarily involved in metabolism, including retinol metabolism, xenobiotic biodegradation and metabolism, and the metabolism of terpenoids and polyketides. The increased abundance of retinol metabolism was also found in lean control rats compared with diabetic fatty rats (105). Terpenoids and polyketides are bioactive substances, and the metabolism of terpenoids and polyketides was significantly increased after weight loss in obese mice (106). Obesity and diabetes are considered to represent low-grade chronic inflammatory states (107). Thus, IAA treatment might act to decrease low-grade chronic inflammatory responses by increasing the activity of the retinol metabolism pathway and the terpenoids and polyketides metabolism pathways. The intestinal microbiota composition plays an important role in xenobiotic biodegradation and metabolism by directly altering the chemical structures of xenobiotics. These changes affect xenobiotic toxicity, biological activity, and bioavailability, which can affect host health (108). Therefore, IAA might promote xenobiotic biodegradation and metabolism in AS mice by regulating the composition of the intestinal microbiota.

In conclusion, the findings of our present study demonstrated the ameliorating effects of IAA in AS model mice induced by PG, which are mediated through the maintenance of intestinal homeostasis and the inhibition of the inflammatory response. The mechanisms of action for IAA against AS involve the regulation of the intestinal microbiota and the activation of the AhR pathway. Therefore, IAA may represent a novel and promising therapy for the treatment of AS, and further research remains necessary to investigate its precise molecular mechanism, which may ultimately result in the development of novel therapies for AS.

## Data Availability Statement

The original contributions presented in the study are publicly available. This data can be found here: NCBI - Bioproject, ID: PRJNA771252.

## Ethics Statement

The animal study was reviewed and approved by the Institutional Animal Care and Use Committee of the Fifth Affiliated Hospital of Sun Yat-sen University. Written informed consent was obtained from the owners for the participation of their animals in this study.

## Author Contributions

JS, LY, and HL conceived and designed the study. JS and KY performed the experiments. TC, ZS, ZC, MW, WZ, and BL analyzed the data and drew the figure. JS wrote the paper and edited the manuscript. LY, KZ, and HL obtained the funding, revised the manuscript, and supervised the whole study. All authors contributed to the article and approved the submitted version.

## Funding

This study was funded by the Natural Science Foundation of China (81572628 and 81372869), Guangdong Province Basic and Applied Basic Research Fund (2019A1515110393), and The Fundamental Research Funds for the Central Universities, Sun Yat-sen University (2021qntd35).

## Conflict of Interest

The authors declare that the research was conducted in the absence of any commercial or financial relationships that could be construed as a potential conflict of interest.

## Publisher’s Note

All claims expressed in this article are solely those of the authors and do not necessarily represent those of their affiliated organizations, or those of the publisher, the editors and the reviewers. Any product that may be evaluated in this article, or claim that may be made by its manufacturer, is not guaranteed or endorsed by the publisher.
